# The Mathematical Foundation of Post-Quantum Cryptography

**DOI:** 10.34133/research.0801

**Published:** 2025-08-26

**Authors:** Chuanming Zong

**Affiliations:** Center for Applied Mathematics, Tianjin University, Tianjin 300072, China.

## Abstract

In 1994, P. Shor discovered quantum algorithms that can break both the RSA cryptosystem and the ElGamal cryptosystem. In 2007, a Canadian company D-Wave demonstrated the first quantum computer. These events and quick further developments have brought a crisis to secret communication. In 2022, the National Institute of Standards and Technology (NIST) announced 4 candidates—CRYSTALS-Kyber, CRYSTALS-Dilithium, Falcon, and Sphincs+—for post-quantum cryptography standards. The first 3 are based on lattice theory and the last on Hash functions. In 2024, NIST announced 3 standards: FIPS 203 based on CRYSTALS-Kyber, FIPS 204 based on CRYSTALS-Dilithium, and FIPS 205 based on Sphincs+. The fourth standard based on Falcon is on the way. It is well known that the security of the lattice-based cryptosystems relies on the hardness of the shortest vector problem (SVP), the closest vector problem (CVP), and their generalizations. In fact, the SVP is a ball packing problem and the CVP is a ball covering problem. Furthermore, both SVP and CVP are equivalent to arithmetic problems for positive definite quadratic forms. There are several books and survey papers dealing with the computational complexity of the lattice-based cryptography for classical computers. However, there is no review article to demonstrate the mathematical foundation of the complexity theory. This paper will briefly introduce post-quantum cryptography and demonstrate its mathematical roots in ball packing, ball covering, and positive definite quadratic forms.

## Mathematical Cryptography

In 1976, W. Diffie and M. E. Hellman [1] proposed the principle of public key cryptography. One year later, the first public key cryptosystem RSA was invented by R. L. Rivest, A. Shamir, and L. Adleman [2]. These events not only inaugurated a new era in secret communication but also marked the birth of mathematical cryptography (see [3,4]), the public key cryptography based on mathematical theories. Since then, several other mathematical cryptosystems have been discovered, including the discrete logarithm cryptosystem invented by T. ElGamal [5] in 1985, the elliptic curve cryptosystem ECC designed by V. S. Miller [6] in 1985 and by N. Koblitz [7] in 1987, respectively, and the lattice-based cryptosystems AD discovered by M. Ajtai and C. Dwork [8] in 1997, GGH invented by O. Goldreich, S. Goldwasser and S. Halevi [9] in 1997, NTRU designed by J. Hoffstein, J. Pipher, and J. H. Silverman [10] in 1998, LWE discovered by O. Regev [11] in 2005, and FHE invented by C. Gentry [12] in 2009. In the past half century, mathematical cryptography has played a crucial role in the modern technology of computers and the internet. At the same time, it has been developed into an active interdisciplinary research field between mathematics and cryptography.

Before Diffie-Hellman, both the enciphering process and the deciphering process of any secret communication used the same secret key. Ciphers of this sort are known as symmetric ciphers. If Bob wants to send a secret message m to Alice, they have to share a secret key k. Bob first scrambles his message m by the key k to a ciphertext c and then sends it through some channel to Alice. When Alice receives the ciphertext c, she uses the secret key k to unscramble it and reconstitute m. During this process, if the communication channel is not secure, their adversary Eve can intercept not only the ciphertext c but also the secret key k and then reconstitute their secret message m.

In the 1970s, when computers and networks were becoming part of daily life, symmetric ciphers were no longer efficient enough for key distribution, key management, and digital signatures. In Diffie and Hellman’s ideal public key cryptosystem, enciphering and deciphering are governed by distinct keys, $k_{e}$ and $k_{d}$, such that computing the decryption key (the private key) $k_{d}$ from the encryption key (the public key) $k_{e}$ is computationally infeasible. All users of a network place their encryption keys in a public directory. Then, the users can encrypt their messages using the receivers’ public keys and decrypt the received messages using their own private keys. We now introduce RSA, NTRU, and LWE as examples, since RSA is the first functioning public key cryptosystem and both NTRU and LWE are crucial for post-quantum cryptography.

### The RSA cryptosystem

First, Alice chooses 2 large primes *p* and *q*, keeps them secret, defines N=pq implying

$$\varphi \left(N\right)=\left(p-1\right)\left(q-1\right),$$ (1)

where $\varphi \left(N\right)$ is Euler’s totient function, and chooses an enciphering exponent *e* satisfying

$$\text{gcd}\left(e,\varphi \left(N\right)\right)=1.$$ (2)

In other words, *e* and $\varphi \left(N\right)$ have no common divisor. Then, she chooses (N,e) as the public key and publishes it. Of course, both Bob and Eve can get it. Second, Bob enciphers his plaintext m by Alice’s public key to the following ciphertext

$$c\equiv m^{e}\;\left(\text{mod}\;N\right)$$ (3)

and sends it to Alice. Third, since Alice knows $\varphi \left(N\right)=\left(p-1\right)$ $\left(q-1\right)$, she can compute *d* satisfying

$$\begin{array}{cc} \mathrm{ed}\;\equiv 1 & \left(\text{mod}\;\varphi \left(N\right)\right) \end{array}$$ (4)

and decipher Bob’s message as

$$\begin{array}{cc} c^{d}\equiv m^{\mathrm{ed}}\equiv m & \left(\text{mod}\;N\right), \end{array}$$ (5)

based on Euler’s formula

$$\begin{array}{cc} m^{\varphi \left(N\right)}\equiv 1 & \left(\text{mod}\;N\right) \end{array}.$$ (6)

In the RSA cryptosystem, besides Euler’s formula, 2 other mathematical results are also crucial. First, when *p* and *q* are known, it is relatively easy to compute the deciphering key *d*. For example, the Euclidean algorithm takes at most $2{\text{log}}_{2}\left(\varphi \left(N\right)\right)+2$ iterations to compute $\text{gcd}\left(e,\varphi \left(N\right)\right)$ and it takes only a small multiple of ${\text{log}}_{2}\left(\varphi \left(N\right)\right)$ steps to compute *d*. On the other hand, without knowledge of *p* and *q*, to factorize the large integer *N* is hard. There are many electronic computer algorithms to factorize large integers. However, none of them are efficient enough to break the RSA cryptosystem. The computational hardness of integer factorization is the security guarantee of the RSA cryptosystem.

### The NTRU cryptosystem

Let *N*, *p*, *q*, $d_{1}$, and $d_{2}$ be suitable integers. Let ${\mathbb{Z}}_{q}$ be the ring of integers modulo *q*, let R, $R_{p}$, and $R_{q}$ be 3 polynomial rings defined by

$$\begin{array}{lll} R & = & \;\mathbb{Z}\left[x\right]/\left(x^{N}-1\right),\\ R_{p} & = & \;{\mathbb{Z}}_{p}\left[x\right]/\left(x^{N}-1\right),\\ R_{q} & = & \;{\mathbb{Z}}_{q}\left[x\right]/\left(x^{N}-1\right), \end{array}$$ (7)

and let $T(d_{1},d_{2})$ denote the set of all polynomials in R that has $d_{1}$ coefficients equal to 1, $d_{2}$ coefficients equal to −1, and all other coefficients equal to 0.

First, Alice and Bob choose a group of public parameters (N,p,q,d) such that both *N* and *p* prime,

gcd(p,q)=gcd(N,q)=1, (8)

and $q>\left(6d+1,\right)p$. Second, Alice chooses $k_{1}\in T(d+1,d)$ and $k_{2}\in T(d,d)$ as private keys, where $k_{1}$ is invertible in both $R_{p}$ and $R_{q}$, computes the inverse $g_{p}$ of $k_{1}$ in $R_{p}$ and the inverse $g_{q}$ of $k_{1}$ in $R_{q}$, computes

$$h=g_{q}k_{2},$$ (9)

and publishes h as the public key. Third, Bob chooses a random r∈T(d,d), encrypts his plaintext $m\in R_{p}$ to

$$\begin{array}{cc} c\equiv p\mathrm{rh}+m & \left(\text{mod}\;q\right), \end{array}$$ (10)

and sends the ciphertext c to Alice. Finally, when Alice receives c, she computes

$$\begin{array}{cc} m^{\circ }\equiv k_{1}c & \left(\text{mod}\;q\right), \end{array}$$ (11)

lifts it to $m^{•}\in R$, and decrypts as

$$\begin{array}{cc} m\equiv g_{p}m^{•} & \left(\text{mod}\;p\right) \end{array}.$$ (12)

More precisely, we have

$$\begin{array}{cc} m^{\circ }=k_{1}c\equiv pk_{1}g_{q}k_{2}r+k_{1}m\equiv pk_{2}r+k_{1}m & \left(\text{mod}\;q\right). \end{array}$$ (13)

Since $k_{1}$, $k_{2}$, r, and m are polynomials of small coefficients, $pk_{2}r+k_{1}m$ has coefficients within (−q/2,q/2) for proper parameters. This means that

$$m^{•}=pk_{2}r+k_{1}m.$$ (14)

### The LWE cryptosystem

Let *n*, *m*, ℓ, *t*, *r*, and *q* be suitable integers and let α be a positive real number. Let ${\mathbb{Z}}_{q}^{n}$ denote the set of vectors $\left(a_{1},a_{2},...,a_{n}\right)$ with $a_{i}\in {\mathbb{Z}}_{q}$, and let ${\mathbb{Z}}_{q}^{n\times \ell }$ denote the set of n×ℓ matrices with entries $a_{\mathrm{ij}}\in {\mathbb{Z}}_{q}$. Furthermore, let $\Psi_{\alpha }$ denote the distribution on ${\mathbb{Z}}_{q}$ obtained by sampling a normal variable with mean 0 and standard deviation $\alpha q/\sqrt{2\pi }$, rounding the result to the nearest integer, and reducing it modulo *q*, let *f* be the function that maps the message space ${\mathbb{Z}}_{t}^{\ell }$ to ${\mathbb{Z}}_{q}^{\ell }$ by multiplying each coordinate by q/t and rounding to the nearest integer, and let $f^{-1}$ denote the inverse of *f*.

First, Alice and Bob choose a group of public parameters $\left(n,m,\ell ,t,r,q,\alpha \right)$. Second, Alice chooses $S\in {\mathbb{Z}}_{q}^{n\times \ell }$ uniformly at random as the private key, takes $A\in {\mathbb{Z}}_{q}^{m\times n}$ uniformly at random, takes $E\in {\mathbb{Z}}_{q}^{m\times \ell }$ by choosing each entry according to $\Psi_{\alpha }$, and chooses (A,P) as the public key, where

P=AS+E. (15)

Third, Bob chooses $a\in {\mathbb{Z}}_{t}^{m}$ uniformly at random and encrypts a message $v\in {\mathbb{Z}}_{t}^{\ell }$ to (u,c), where $u=A^{\prime }a$ and

$$c=P^{\prime }a+f\left(v\right).$$ (16)

Finally, when Alice receives (u,c), she decrypts it by her secret key *S* as

$$v=f^{-1}\left(c-S^{\prime }u\right).$$ (17)

Lattice is a mathematical concept introduced by Gauss at the beginning of the 19th century and further developed by Minkowski and many others (see [13,14]). Let $a_{1},a_{2},\ldots ,a_{n}$ be *n* linearly independent vectors in the *n*-dimensional Euclidean space $E^{n}$. We call

$$\Lambda =\left\{z_{1}a_{1}+z_{2}a_{2}+\ldots +z_{n}a_{n}:\;z_{i}\in \mathbb{Z}\right\}$$ (18)

an *n*-dimensional lattice and call $\left\{a_{1},a_{2},...,a_{n}\right\}$ a basis of the lattice Λ.

At the first glance, both NTRU and LWE have nothing to do with lattice. In fact, both of them can be reformulated in lattice and their security depends on the computational complexity of some lattice problems (see [15]).

## Post-Quantum Cryptography

The classical computer is based on the laws of electronics. Its fundamental unit of information is the binary digit (bit) 0 or 1. Sequences of bits are manipulated by Boolean logic gates, and a succession of gates yields a computation.

### Quantum Turing machine

At the beginning of the 1980s, Y. I. Manin, P. Benioff, R. Feynman, and D. Deutsch started investigating the possibility of creating a computer based on the laws of quantum mechanics (see [16]). In particular, Deutsch introduced the quantum Turing machine and quantum circuits in 1985.

A quantum computer operates on quantum bits (or qubits). The state of a qubit can be represented as

$$\alpha_{1}|0\rangle +\alpha_{2}|1\rangle ,$$ (19)

where $|0\rangle$ is its ground state, $|1\rangle$ is its excited state, and $\alpha_{i}$ are complex numbers satisfying ${\left|\alpha_{1}\right|}^{2}+{\left|\alpha_{2}\right|}^{2}=1.$ In a system of *n* qubits, let $|s_{i}\rangle =|s_{1}^{i}s_{2}^{i}\ldots s_{n}^{i}\rangle$ denote the $2^{n}$ basis states with $s_{j}^{i}\in \{0,1\}$, the superposition of states can be represented as

$$\sum_{i=1}^{2^{n}}\alpha_{i}|s_{i}\rangle ,$$ (20)

where $\alpha_{i}$ are complex numbers satisfying $\sum {\left|\alpha_{i}\right|}^{2}=1$, and ${\left|\alpha_{i}\right|}^{2}$ represents the possibility of the system yield state $|s_{i}\rangle$. The quantum computer manipulates qubits via quantum logic gates to process computations. A quantum logic gate will change one superposition of states to one other superposition of states by a unitary transformation, where unitary means that the conjugate transpose of the transformation matrix is equal to its inverse. For example, suppose a quantum computer of 3 qubits is in the superposition of states

$$\;\frac{1}{2}|000\rangle -\frac{1}{2}|010\rangle +\frac{1}{2}|101\rangle -\frac{1}{2}|111\rangle \;$$ (21)

and the logic gate changes the last 2 qubits of the state by

$$\begin{array}{c} 00\\ 01\\ 10\\ 11 \end{array}\to \left(\begin{array}{rrrr} \frac{1}{2} & \frac{1}{2} & \frac{1}{2} & \frac{1}{2}\\ \frac{1}{2} & \frac{i}{2} & -\frac{1}{2} & -\frac{i}{2}\\ \frac{1}{2} & -\frac{1}{2} & \frac{1}{2} & -\frac{1}{2}\\ \frac{1}{2} & -\frac{i}{2} & -\frac{1}{2} & \frac{i}{2} \end{array}\right)\begin{array}{c} 00\\ 01\\ 10\\ 11. \end{array}$$ (22)

Then, the computer will go to the superposition of states

$$\;\frac{1}{2}|001\rangle +\frac{1}{2}|011\rangle +\frac{i}{2}|101\rangle -\frac{i}{2}|111\rangle .$$ (23)

Since the state of the output of a quantum computer can be a coherent superposition of states corresponding to different solutions of a problem, it may allow many computations to be done simultaneously and quickly (see [17]).

### Quantum computing

In the early 1990s, when the quantum computer was not yet born, Deutsch, R. Jozsa, Shor, and L. Grover started to explore quantum computing (see [16]). First, Deutsch and Jozsa [18] presented a problem that can be solved by a quantum computer with certainty in polynomial time, which is exponentially less time than any classical deterministic computer, and less than the expected time of any classical stochastic computer.

Almost at the same time, Shor [19] discovered polynomial time quantum algorithms to deal with the discrete logarithm problem and the factorization problem. Assume that 0≤ a<q and

$$a=\sum_{i=0}^{k-1}\alpha_{i}2^{i}$$ (24)

is the binary representation of *a*. Then, he defines the state $|a\rangle =|\alpha_{k-1}\alpha_{k-2}\cdots \alpha_{0}\rangle$ and introduces the following unitary transformation:

$$|a\rangle \to \frac{1}{q^{1/2}}\sum_{b=0}^{q-1}\exp\left(2\text{πiab}/q\right)|b\rangle .$$ (25)

This transformation, as a quantum logic gate, plays a key role in his algorithms. A decade later, J. Proos and C. Zalka [20] succeeded in modifying Shor’s discrete logarithm quantum algorithm for elliptic curves. It follows that once there is a functioning quantum computer, Shor’s algorithms could break the RSA cryptosystem, the ElGamal cryptosystem, and the ECC cryptosystem. Over the years, several improvements to Shor’s algorithms have been discovered. For example, the one was announced by Regev [21] in 2023.

### Quantum computer

In 1998, the first quantum computer models appeared at Oxford University, IBM’s Almaden Research Center, and Los Alamos. In 2007, a Canadian company D-Wave demonstrated the Orion system, a 16-qubit quantum annealing processor, running 3 different applications at the Computer History Museum in Mountain View, California. This marked the first public demonstration of a quantum computer. In 2011, D-Wave announced D-Wave One, operating on a 128-qubit chipset using quantum annealing to solve optimization problems. In the following years, several companies developed gate model quantum machines, including Google, IBM, Intel, and Rigetti. Gate model quantum computers use gates similar in concept to classical computers but with vastly different logic and architecture. By 2020, there were about a hundred working quantum computers worldwide.

In 2001, a group of researchers at IBM successfully applied Shor’s algorithm to factorize 15, using nuclear magnetic resonance. In 2019, the numbers 15, 21, and 35 were factorized by applying Shor’s algorithm on a 6-qubit IBM quantum processor (see [22]).

### Post-quantum cryptography

As larger and larger quantum computers are built, cryptosystems such as RSA, ElGamal, and ECC will no longer be secure, so post-quantum cryptography will be critical for the future of secret communication.

In 2006, the first international workshop on post-quantum cryptography took place at the Katholieke Universiteit Leuven. Since then, post-quantum cryptography has gradually become an important research branch of cryptography. In particular, it has become a focus topic of CRYPTO, EUROCRYPT, and ASIACRYPT.

In 2016, the National Institute of Standards and Technology (NIST) launched a global project to solicit and select a handful of encryption algorithms with the ability to resist quantum computer attacks. On 2022 July 5, after 3 rounds of competition and selection, NIST announced 4 algorithms that will underpin its future cryptography standards. They include one algorithm (CRYSTALS-Kyber) for general encryption and key establishment purposes and 3 (CRYSTALS-Dilithium, Falcon, and Sphincs+) for digital signatures (see [23–25]). On 2024 August 13, the agency announced 3 post-quantum cryptography standards: FIPS 203 based on CRYSTALS-Kyber, FIPS 204 based on CRYSTALS-Dilithium, and FIPS 205 based on Sphincs+. The fourth standard based on Falcon is on the way. On 2024 November 12, NIST published the guideline “Transition to post-quantum cryptography standards”, which lists detailed route and time table. In fact, many high-tech companies and institutions have already completed the transition.

It is well known that both CRYSTALS-Kyber and CRYSTALS-Dilithium are based on LWE, Falcon is based on NTRU, and Sphincs+ is based on Hash functions. Both NTRU and LWE are lattice-based cryptosystems. Lattice-based cryptography was born more or less at the same time of Shor’s quantum algorithms for the discrete logarithm problem and the factorization problem (see [26–28]). It has been explored as a key candidate for post-quantum cryptography ever since.

## The Shortest Vector Problem and the Closest Vector Problem

No one can predict the future of the post-quantum cryptography. Currently, a decisive role is played by lattice-based cryptosystems. No matter how different in form, the security of all known lattice-based cryptosystems and algorithms relies on the computational complexity of the following 2 problems and their variations:

**The shortest vector problem (SVP):** Find a shortest nonzero vector in an *n*-dimensional lattice Λ, i.e., find a nonzero vector v∈Λ that minimizes the Euclidean norm $\left\|v\right\|$.

**The closest vector problem (CVP):** Given a vector $x\in E^{n}$ that is not in Λ, find a vector v∈Λ that is closest to x, i.e., find a vector v∈Λ that minimizes the Euclidean norm $\left\|v-x\right\|$.

In fact, the security of all AD , NTRU, and LWE depends on the complexity of SVP and its variations, and the security of GGH and NTRU is based on the complexity of CVP and its approximation (see [15,27]).

### Complexity theory of classical computer

A Turing machine M runs in time *t*(*n*) if, for every input string s of length *n* over some fixed input alphabet, $M\left(s\right)$ halts after at most *t*(*n*) steps. Efficient computation with a Turing machine means that it halts in polynomial time in the size of the input, i.e., the Turing machine runs in time $t\left(n\right)=a+n^{b}$ for some constants *a* and *b* independent of *n*.

A decision problem consists of deciding whether the input string satisfies some specified property or not. The class of decision problems that can be solved by a deterministic Turing machine in polynomial time is called P. The class of decision problem that can be solved by a nondeterministic Turing machine in polynomial time is called NP. Clearly, we have P⊆NP. It is widely believed that P≠NP, i.e., there are NP problems that cannot be solved in deterministic polynomial time. In fact, to prove or disprove P=NP is a fundamental problem in both mathematics and computer science.

Let $P_{1}$ and $P_{2}$ be 2 decision problems consisting of strings of alphabet. A reduction from $P_{1}$ to $P_{2}$ is a polynomial time computable function *f* such that $s\in P_{1}$ if and only if $f\left(s\right)\in P_{2}$. Clearly, if $P_{1}$ reduces to $P_{2}$ and $P_{2}$ can be solved in polynomial time, then $P_{1}$ can also be solved in polynomial time. A decision problem *P* is NP-hard if any other NP problem *Q* reduces to *P*. If *P* is also in NP, then *P* is NP-complete. Evidently, if a problem *P* is NP-hard, then *P* cannot be solved in polynomial time unless P=NP.

### The complexity of SVP for the classical computer

First, a lattice may have many shortest vectors. It is easy to see that the integer lattice ${\mathbb{Z}}^{n}$ has 2*n* shortest vectors. It is known that the 8-dimensional $E_{8}$ lattice has 240 shortest vectors and the 24-dimensional Leech lattice has 196,560 shortest lattice vectors. In general, an *n*-dimensional lattice Λ has at most

$$2^{0.401n\left(1+o\left(1\right)\right)}$$ (26)

shortest vectors (see [14]). However, lattice-based cryptography uses random lattices rather than a particular one, so the following result is pertinent.

**Theorem 3.1 (Södergren** [29]**).** In $E^{n}$, n≥2, a random lattice has exactly one pair $\left(\pm v\right)$ of shortest nonzero vectors, i.e., if we randomly pick a lattice, the probability of it having only one pair of shortest lattice vectors is one.

It is interesting to notice that Theorem 3.1 was proved only in 2010, much later than it was applied in lattice-based cryptography. Obviously, it has been taken for granted by cryptographers. Nevertheless, if Theorem 3.1 were not true, the SVP-based cryptosystems would be impossible.

Usually, lattices are given by their bases. One may intuitively believe that the bases should contain some short lattice vector. In fact, this is far from the truth. For example, let Λ be the integer lattice ${\mathbb{Z}}^{2}$, let *m* be a large integer, and define $a_{1}=\left(1,\;m+1\right)$ and $a_{2}=-\left(1,\;m\right)$. It can be verified that $\left\{a_{1},\;a_{2}\right\}$ is a basis of Λ and

$$\left\|a_{1}\right\|\geq \left\|a_{2}\right\|=\sqrt{1+m^{2}}.$$ (27)

In other words, both vectors of a basis of Λ can be arbitrarily long. Nevertheless, the length of the shortest vectors of a lattice Λ can be bounded in terms of its determinant $\det\left(\Lambda \right)$. In 1891, Minkowski obtained the following fundamental result about the length of the shortest lattice vector.

**Theorem 3.2.** Every lattice Λ of dimension *n* contains a nonzero vector v satisfying

$$\left\|v\right\|\leq \left(\sqrt{2/\pi e}+o\left(1\right)\right)\sqrt[{n}]{\text{det}\left(\Lambda \right)}\sqrt{n}.$$ (28)

This result tells us the approximate range of the shortest lattice vectors. It can be regarded as the first cornerstone of the lattice-based cryptography.

At the beginning of the 1980s, about 2 decades before lattice-based cryptography was born, people started to study the computational complexity of lattices. In 1981, P. van Emde Boas [30] made the following conjecture.

**Conjecture 3.1.** The SVP is NP-hard.

In the same paper, he proved that the SVP in the $L_{\infty }$ norm is indeed NP-hard. However, 40 years later, the Euclidean case is still open today. Meanwhile, research has turned toward randomized reduction and approximation. Unlike deterministic reduction, randomized reduction allows the mapping function to be computable in polynomial time by a probabilistic algorithm. (A probabilistic Turing machine is a nondeterministic Turing machine that chooses between the available transitions at each point according to some probability. A quantum computer is another model of computation that is inherently probabilistic.) Therefore, the output of the reduction is only required to be correct with sufficiently high probability. In 1997, Ajtai [31] proved the following theorem.

**Theorem 3.3.** The SVP is NP-hard under randomized reduction.

In fact, even approximating the shortest vector is not easy. In 1998, D. Micciancio improved Ajtai’s theorem, showing that approximating the shortest vector within a factor $\sqrt{2}$ under randomized reduction is NP-hard. In 2005, S. Khot [32] proved the following theorem.

**Theorem 3.4.** To approximate the shortest vector of an *n*-dimensional lattice within any constant factor *c* under randomized reduction is NP-hard.

All Ajtai, Micciancio, and Khot’s works deal with general $L_{p}$ norms. For simplicity, we only concentrate on the Euclidean case. Theorem 3.4 has been further extended by I. Haviv, Regev, and others.

In 2004, Ajtai [33] introduced a new problem, called the short integer solution (SIS) problem, over random q-ary lattices. He proved that, under certain hypotheses, solving SIS over a lattice chosen randomly from an easily samplable distribution is at least as hard as approximating the SVP for any lattice.

### The complexity of CVP for the classical computer

In 1981, when he proposed Conjecture 3.1, van Emde Boas proved that CVP is NP-hard. On the other hand, it can be shown that CVP is in NP. Thus, we have the following theorem.

**Theorem 3.5.** The CVP is NP-complete.

As with the SVP, there are many complexity results about approximating the CVP. We cite one of them here as an example.

**Theorem 3.6 (Dinur, Kindler, Raz and Safra** [34]**).** To approximate the closest vector of an *n*-dimensional lattice to a given point of $E^{n}$ within a factor $n^{c/\text{log}\text{log}n}$, where *c* is some absolute constant, is NP-hard.

It was conjectured by L. Babai in 1986 that the SVP is not harder than the CVP. In 1999, this conjecture was proved by Goldreich et al. [35]. On the other hand, in practice, a CVP in dimension *n* can usually be transformed into solving an SVP in dimension n+1; so, for cryptographic purposes, they tend to be of roughly equal difficulty.

**Theorem 3.7.** There is an approximation-preserving polynomial time reduction from the SVP to the CVP.

### The Lenstra–Lenstra–Lovász algorithm

Since every pair of bases of a lattice is connected by a unimodular matrix, when the initial basis of the lattice is not very good (for example, from the perspective of orthogonality), one may hope to reduce it to a good one. It is easy to show that, if $v_{1}$ is one of the shortest vectors of the lattice, it has a basis with $v_{1}$ as one of the *n* generators. Many great mathematicians have made contributions in reduction theory, including Lagrange, Gauss, Hermite, Minkowski, Voronoi, Korkin, and Zolotarov (see [14,36]). Nevertheless, in higher dimensions, finding or even approximating the shortest vector turns out to be extremely hard. In 1982, A. K. Lenstra, H. W. Lenstra Jr., and L. Lovász [37] proposed an algorithm (known as the LLL algorithm), which can not only efficiently approximate the shortest vector of a lattice but also approximate the closest vector.

**Theorem 3.8.** Let Λ be an *n*-dimensional integer lattice, i.e., $\Lambda \subseteq {\mathbb{Z}}^{n}$, and let $\ell \left(\Lambda \right)$ denote the length of the shortest nonzero vector of Λ. The LLL algorithm can find a nonzero lattice vector v∈Λ in polynomial time satisfying

$$\left\|v\right\|\leq {\left(2/\sqrt{3}\right)}^{n}\ell \left(\Lambda \right).$$ (29)

**Theorem 3.9 (Babai** [38]**).** There are polynomial time algorithms that solve the CVP within a factor $2{\left(2/\sqrt{3}\right)}^{n}$. In other words, for any $x\in E^{n}$ one can find a lattice vector v∈Λ satisfying

$$\left\|x-v\right\|\leq 2{\left(2/\sqrt{3}\right)}^{n}d\left(x,\;\Lambda \right).$$ (30)

In both Theorem 3.8 and Theorem 3.9, the approximation factors are exponential in the dimensions. Over the years, many efforts have been made to improve the approximation factors, such as the BKZ algorithm proposed in 1987 by C.-P. Schnorr and R. Kannan (see [39]). Nevertheless, no real progress has been achieved. Essentially, all these algorithms are based on various types of basis reductions, which will be introduced in the last section. 

SVP and CVP have several variants and generalizations that are useful in lattice-based cryptosystems as well, such as SVP_γ_, CVP*_γ_*, GapSVP, GapCVP, the shortest basis problem (SBP), the quasi-orthogonal basis problem (QOBP), the successive minima problem (SMP), the shortest independent vector problem (SIVP), the shortest diagonal problem (SDP), and the densest sublattice problem (DSP) (see [13,15,27,28,40]). For example, let Λ be an *n*-dimensional lattice and let *k* be a given positive number, the GapSVP with approximation factor $\gamma \left(n\right)$ asks to decide whether $\ell \left(\Lambda \right)\leq k$ or $\ell \left(\Lambda \right)>\gamma \left(n\right)k$.

### The complexity of SVP and CVP for the quantum computer

Since the birth of Shor’s quantum algorithms for discrete logarithms and factoring in 1994, in particular since the NIST initiated the post-quantum cryptography competition in 2016, people have tried hard to search for efficient quantum computing algorithms for the SVP and the CVP, or tried to prove that there is no such algorithm. Up to now, none of this effort has succeeded. This failure led to the following conjectures.

**Conjecture 3.2.** There is no polynomial time quantum algorithm that can approximate the SVP within a polynomial factor.

**Conjecture 3.3.** There is no polynomial time quantum algorithm that can approximate the CVP within a polynomial factor.

If Conjectures 3.2 and 3.3 are correct, they will provide evidence for the security of lattice-based cryptosystems in the quantum computing era. For the updated computational results, we refer the readers to [41].

## Ball Packing and Ball Covering

Let $B^{n}$ denote the *n*-dimensional unit ball $\left\{x:\;\sum x_{i}^{2}\leq 1\right\}$ in $E^{n}$ and let *X* denote a discrete set of points in $E^{n}$. We call $B^{n}+X=\left\{B^{n}+x_{i}\;:\;x_{i}\in X\right\}$ a ball packing (in discrete geometry, it is called sphere packing rather than ball packing) if the interiors of the balls are disjoint. In particular, we call it a lattice ball packing if *X* is a lattice. Let $\delta \left(B^{n}\right)$ denote the density of the densest ball packings in $E^{n}$ and let $\delta^{*}\left(B^{n}\right)$ denote the density of the densest lattice ball packings. Clearly, we have

$$\delta^{*}\left(B^{n}\right)\leq \delta \left(B^{n}\right).$$ (31)

**Fig. 1.**
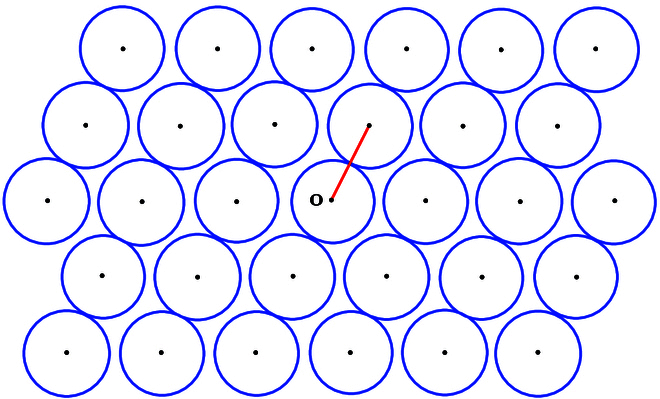
SVP in ball packing. The balls of the radii of half the length of the shortest lattice vectors form a lattice packing.

Assume that Λ is an *n*-dimensional lattice in $E^{n}$. Let $\ell \left(\Lambda \right)$ denote the length of the shortest nonzero vectors of Λ and take $r=\ell \left(\Lambda \right)/2$. It is easy to see that ${\mathrm{rB}}^{n}+\Lambda$ is a lattice ball packing in $E^{n}$ (see Fig. 1). Then, the SVP can be reformulated in terms of ball packing.

**SVP in ball packing.** For a given n-dimensional lattice Λ, find the largest number *r* such that ${\mathrm{rB}}^{n}+\Lambda$ is a ball packing and the corresponding balls that touch ${\mathrm{rB}}^{n}$ at its boundary.

In fact, based on the previous discussion, one can deduce the following connection between the length $\ell \left(\Lambda \right)$ of the shortest nonzero vector of a lattice Λ and the ball packing densities $\delta^{*}\left(B^{n}\right)$ and $\delta \left(B^{n}\right)$.

**Theorem 4.1.** Let Λ be an *n*-dimensional lattice and let $\omega_{n}$ denote the volume of $B^{n}$. We have

$$\ell \left(\Lambda \right)\leq 2\sqrt[{n}]{\text{det}\left(\Lambda \right)\cdot \delta^{*}\left(B^{n}\right)/\omega_{n}}\leq 2\sqrt[{n}]{\text{det}\left(\Lambda \right)\cdot \delta \left(B^{n}\right)/\omega_{n}}.$$ (32)

Ball packing, including the study of $\delta \left(B^{n}\right)$ and $\delta^{*}\left(B^{n}\right)$, is a classic subject in mathematics. It has been studied by many prominent mathematicians including Kepler, Newton, Gauss, and Minkowski (see [14]). However, our knowledge in this field is still very limited.

In 1594, T. Harriot discovered the face-centered cubic lattice ball packing in $E^{3}$ and determined that its density is $\pi /\sqrt{18}=0.74\cdots$. However, he was not able to prove that the density is the maximum. Then, he told his discovery to Kepler. In 1611, Kepler made the following conjecture: *The density of the densest ball packing in* $E^{3}$ *is* $\pi /\sqrt{18}$. *In other words*,

$$\delta \left(B^{3}\right)=\frac{\pi }{\sqrt{18}}.$$ (33)

In 1694, Newton and D. Gregory discussed the following problem: *Can* 13 *unit balls in* $E^{3}$ *be brought into contact with a fixed one*? These 2 natural and simple sounding problems initiated ball packing as a field of mathematical research. Some key results about $\delta^{*}\left(B^{n}\right)$ and $\delta \left(B^{n}\right)$ are summarized in Table 1 (see [14,42,43]).

**Table 1.** Known results about ball packing densities

n	$\delta^{*}\left(B^{n}\right)$	Author Date	$\delta \left(B^{n}\right)$	Author Date
2	$\frac{\pi }{\sqrt{12}}$	Lagrange 1173	$\frac{\pi }{\sqrt{12}}$	Thue 1892
3	$\frac{\pi }{\sqrt{18}}$	Gauss 1831	$\frac{\pi }{\sqrt{18}}$	Hales 2005
4	$\frac{\pi^{2}}{16}$	Korkin, Zolotarev 1872	??	??
5	$\frac{\pi^{2}}{15\sqrt{2}}$	Korkin, Zolotarev 1877	??	??
6	$\frac{\pi^{3}}{48\sqrt{3}}$	Blichfeldt 1925	??	??
7	$\frac{\pi^{3}}{105}$	Blichfeldt 1926	??	??
8	$\frac{\pi^{4}}{384}$	Blichfeldt 1934	$\frac{\pi^{4}}{384}$	Viazovska 2017
24	$\frac{\pi^{12}}{12!}$	Cohn, Kumar 2009	$\frac{\pi^{12}}{12!}$	Cohn, Kumar, Miller, Radchenko, Viazovska 2017

Besides Theorem 4.1, reduction methods to determine the values of $\delta^{*}\left(B^{n}\right)$ are useful in algorithms for SVP and CVP. For example, the Korkin–Zolotarov reduction is employed in the block Korkin–Zolotarov algorithm developed by Schnorr [39] in 1987.

In general dimensions, we have

$${\mathrm{cn}}^{2}2^{-n}\leq \delta^{*}\left(B^{n}\right)\leq \delta \left(B^{n}\right)\leq 2^{-0.599n\left(1+o\left(1\right)\right)}$$ (34)

for a suitable positive constant *c*, where a weaker lower bound was first proved by Minkowski in 1905, then improved and generalized by E. Hlawka, C. L. Siegel, H. Davenport, C. A. Rogers, W. M. Schmidt, B. Klartag, and others (see [44]), and the upper bound was proved by G. A. Kabatjanski and V. I. Levenštein in 1978 (see [14]). Clearly, the upper bound and Theorem 4.1 have the following corollary, which is an improvement of Theorem 3.2.

**Corollary 4.1.** Every lattice Λ of dimension *n* contains a nonzero vector v satisfying

$$∥v∥\leq \left(\pi^{-0.5}e^{-0.5}2^{-0.099}+o\left(1\right)\right)\sqrt[{n}]{\text{det}\left(\Lambda \right)}\sqrt{n}.$$ (35)

There are hundreds of papers on ball packing, employing methods and tools from various fields of mathematics. As well, there are many fascinating open problems on ball packing. Here, we list 2 of them as examples.

**Problem 4.1.** Determine the asymptotic orders of $\delta^{*}\left(B^{n}\right)$ and $\delta \left(B^{n}\right)$, if they exist.

**Problem 4.2.** Is there a dimension *n* satisfying

$$\delta^{*}\left(B^{n}\right)\neq \delta \left(B^{n}\right)?$$ (36)

Clearly, a solution to Problem 4.1 will provide further improvement of Theorem 4.1 and better understanding of SVP. Similar to the ball case, one can define and study lattice packing of any centrally symmetric convex body, which corresponds to the SVP in a metric linear space.

Assume that Λ is an *n*-dimensional lattice in $E^{n}$. For every point $x\in E^{n}$, we define the distance between x and its closest lattice point v∈Λ as $d\left(x,\;\Lambda \right)$. Then, we define

$$\rho \left(\Lambda \right)=\underset{x\in E^{n}}{\text{max}}d\left(x,\;\Lambda \right).$$ (37)

**Fig. 2.**
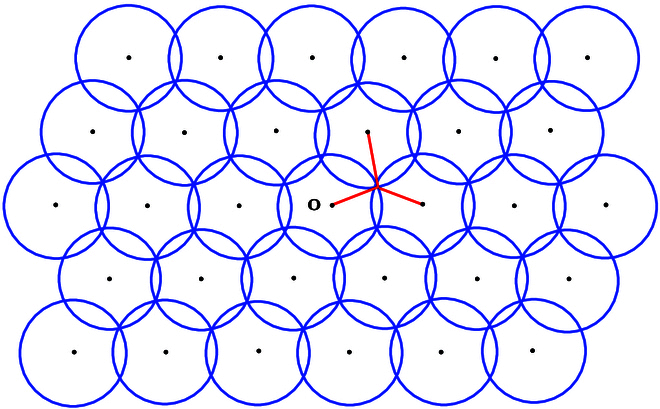
CVP in the ball covering. The balls of radii of the maximum distance between a point to its closest lattice vectors form a lattice covering.

It is easy to see that $\rho \left(\Lambda \right)B^{n}+\Lambda$ is a covering of $E^{n}$ (see Fig. 2). In fact, $\rho \left(\Lambda \right)$ is the smallest radius ρ such that $\rho B^{n}+\Lambda$ is a covering of $E^{n}$.

**CVP in ball covering.** Given an *n*-dimensional lattice Λ, find the smallest number ρ such that $\rho B^{n}+\Lambda$ is a covering of $E^{n}$. For any $x\in E^{n}$, find a lattice point $v\in \rho B^{n}+x$.

Clearly, finding a lattice point $v\in \rho B^{n}+x$ is slightly simpler than the CVP. However, this covering model can illustrate the fundamental difficulty of the CVP. First, unlike Theorem 3.2 and Theorem 4.1, there is no upper bound for $\rho \left(\Lambda \right)$ in terms of $\text{det}\left(\Lambda \right)$ and *n*. Let *m* be a large integer, take $a_{1}=\left(m,\;0\right)$ and $a_{2}=\left(0,1/m\right)$, and define Λ to be the 2-dimensional lattice generated by $a_{1}$ and $a_{2}$. Then, we have $\text{det}\left(\Lambda \right)=1$. If $x=\left(m/2,1/2m\right)$, one can easily deduce that

$$\rho \left(\Lambda \right)=d\left(x,\Lambda \right)=\frac{1}{2}\;\sqrt{m^{2}+1/m^{2}}.$$ (38)

Apparently, $\rho \left(\Lambda \right)$ can not be bounded from above just in terms of $\det\left(\Lambda \right)$.

Let $\theta \left(B^{n}\right)$ denote the density of the thinnest ball covering of $E^{n}$ and let $\theta^{*}\left(B^{n}\right)$ denote the density of the thinnest lattice ball covering of $E^{n}$. As a counterpart to Theorem 4.1, we have the following relation between $\rho \left(\Lambda \right)$ and $\theta^{*}\left(B^{n}\right)$.

**Theorem 4.2.** Let Λ be an *n*-dimensional lattice and let $\omega_{n}$ denote the volume of $B^{n}$. We have

$$\rho \left(\Lambda \right)\geq \sqrt[{n}]{\text{det}\left(\Lambda \right)\cdot \theta^{*}\left(B^{n}\right)/\omega_{n}}\geq \sqrt[{n}]{\text{det}\left(\Lambda \right)\cdot \theta \left(B^{n}\right)/\omega_{n}}.$$ (39)

Ball covering, in certain sense, is regarded as a dual concept of ball packing. In fact, they are not much related. Up to now, the known exact results about $\theta \left(B^{n}\right)$ and $\theta^{*}\left(B^{n}\right)$ are summarized in Table 2.

**Table 2.** Known results about ball covering densities

*n*	$\theta^{*}\left(B^{n}\right)$	Author Date	$\theta \left(B^{n}\right)$	Author Date
2	$\frac{2\pi }{3\sqrt{3}}$	Kersshner 1939	$\frac{2\pi }{3\sqrt{3}}$	Kersshner 1939
3	$\frac{5\sqrt{5}\pi }{24}$	Bambah 1954	??	??
4	$\frac{2\pi^{2}}{5\sqrt{5}}$	Delone, Ryskov 1963	??	??
5	$\frac{245\sqrt{35}\pi^{2}}{3888\sqrt{3}}$	Ryskov, Baranovskii 1975	??	??

In general dimensions, there is a constant *c* such that

$$\left(1+o\left(1\right)\right)\frac{n}{\sqrt{e^{3}}}\leq \theta \left(B^{n}\right)\leq \theta^{*}\left(B^{n}\right)\leq \mathrm{cn}{\left({\text{log}}_{e}n\right)}^{{\text{log}}_{2}\sqrt{2\pi e}},$$ (40)

where the lower bound was achieved by H. S. M. Coxeter, L. Few, and Rogers in 1959, and the upper bound was discovered by Rogers in 1959 (see [21,45,46]). Clearly, the lower bound and Theorem 4.2 have the following corollary, which in certain sense shows the complexity of CVP.

**Corollary 4.2.** Let Λ be an *n*-dimensional lattice. We have

$$\rho \left(\Lambda \right)\geq \left({\left(2\pi e\right)}^{-0.5}+o\left(1\right)\right)\sqrt[{n}]{\text{det}\left(\Lambda \right)}\sqrt{n}.$$ (41)

Corollary 4.1 and Corollary 4.2 together provide an explanation for Theorem 3.7, i.e., the CVP is harder than the SVP.

One may realize that there are very few concrete results on ball covering in the past half a century, particularly compared to ball packing. It is fascinating to notice that, unlike the packing case, the thinnest lattice ball covering in $E^{8}$ is not achieved by the $E_{8}$ lattice. At least, the $A_{8}^{*}$ lattice provides a ball covering with a density thinner than the $E_{8}$ lattice. Therefore, the following problem is important and perhaps very challenging.

**Problem 4.3.** Determine the values of $\theta^{*}\left(B^{8}\right)$, $\theta \left(B^{8}\right)$, $\theta^{*}\left(B^{24}\right)$, and $\theta \left(B^{24}\right)$.

### Two bridges connecting SVP and CVP

Let $L_{n}$ denote the family of all *n*-dimensional lattices. In 1950, Rogers defined and studied

$$\phi^{*}\left(B^{n}\right)=\underset{\Lambda \in L_{n}}{\text{min}}\frac{2\rho \left(\Lambda \right)}{\ell \left(\Lambda \right)},$$ (42)

where $\ell \left(\Lambda \right)$ is the length of the shortest nonzero vectors of Λ and $\rho \left(\Lambda \right)$ is the maximum distance between a point $x\in E^{n}$ and its closest lattice point. They are known as Rogers’ constants.

From the intuitive point of view, one may think that $\phi^{*}\left(B^{n}\right)$ can be arbitrarily large when n→∞. Surprisingly, Rogers proved by a reduction method that

$$\phi^{*}\left(B^{n}\right)\leq 3$$ (43)

holds in every dimension. In 1972, via mean value techniques developed by Rogers and Siegel, G. L. Butler improved Rogers’ upper bound to

$$\phi^{*}\left(B^{n}\right)\leq 2+o\left(1\right).$$ (44)

It follows from Rogers’ upper bound that, for many *n*-dimensional lattices, the longest distance in CVP is only a constant multiple of the length of the SVP. In recent years, this idea has been applied to cryptographic analysis by Micciancio [47] and others.

The constant $\phi^{*}\left(B^{n}\right)$ has a couple of different interpretations. For example, $\phi^{*}\left(B^{n}\right)$ is the largest number such that every lattice ball packing $B^{n}+\Lambda$ has a hole into which one can put a ball of radius $\phi^{*}\left(B^{n}\right)-1$. In the 1980s, several mathematicians studied $\phi^{*}\left(B^{n}\right)$ from different respects. Up to now, the known exact results are listed in Table 3.

**Table 3.** Known results about Rogers’ constants

*n*	2	3	4	5
$\phi^{*}\left(B^{n}\right)$	$2/\sqrt{3}$	$\sqrt{5/3}$	$\sqrt{2\sqrt{3}}\left(\sqrt{3}-1\right)$	$\sqrt{\frac{3}{2}+\sqrt{\frac{13}{6}}}$
Author Date		Boroczky 1986	Horvath 1982	Hovarth 1986

Just like the ball covering case, there are many important open problems about $\phi^{*}\left(B^{n}\right)$. We list 2 of them here as examples.

**Problem 4.4.** Determine the values of $\phi^{*}\left(B^{8}\right)$ and $\phi^{*}\left(B^{24}\right)$, and their corresponding lattices.

**Problem 4.5.** Is there a dimension *n* such that

$$\phi^{*}\left(B^{n}\right)\geq 2?$$ (45)

What is known about the Leech lattice supports the conjecture that $\phi^{*}\left(B^{24}\right)=\sqrt{2}$. If one can improve Butler’s upper bound to $\phi^{*}\left(B^{n}\right)\leq 2-c$, where *c* is a positive constant, the lower bound for $\delta^{*}\left(B^{n}\right)$ will be improved to

$$\delta^{*}\left(B^{n}\right)\geq {\left(2-c\right)}^{-n}.$$ (46)

If a dimension *n* can be found such that $\phi^{*}\left(B^{n}\right)\geq 2$, then

$$\delta^{*}\left(B^{n}\right)\neq \delta \left(B^{n}\right),$$ (47)

which would solve Problem 4.2. It is easy to see that $\phi^{*}\left(B^{n}\right)$ can be generalized from the ball to arbitrary centrally symmetric convex bodies. For more on $\phi^{*}\left(B^{n}\right)$ and its generalizations, we refer to [48–50].

There is another important notion that is closely related to both the SVP and the CVP, the Dirichlet–Voronoi cell of Λ:

$$\begin{array}{c} D=\left\{x:\;\langle x,\;v\rangle \;\leq \;\frac{1}{2}\;\langle v,\;v\rangle \;\text{for}\;\mathrm{all}\;v\in \;\Lambda \backslash \;\left\{o\right\}\;\right\}. \end{array}$$ (48)

**Fig. 3.**
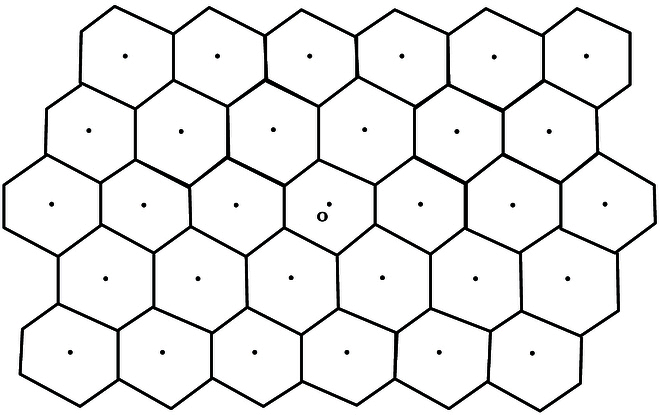
CVP in D–V cell. The Dirichlet–Voronoi cells form a lattice tiling.

Roughly speaking, *D* is the set of points that are closer to the origin than any other lattice point. Clearly, *D* is a centrally symmetric polytope such that D+Λ is a tiling of $E^{n}$ (see Fig. 3).

Furthermore, one can deduce that

$$\ell \left(\Lambda \right)=2\text{min}\left\{d\left(o,\;F\right):\;F\;\text{is}\;a\;\text{facet}\;\text{of}\;D\;\right\}$$ (49)

and

$$\rho \left(\Lambda \right)=\text{max}\left\{\;∥\;v\;∥:\;v\;\text{is}\;a\;\text{vertex}\;\text{of}\;D\;\right\}.$$ (50)

Therefore, the Dirichlet–Voronoi cell of a lattice encodes information about both SVP and CVP. In fact, the CVP can be reformulated as:

**CVP in D–V cell.** Let Λ be an *n*-dimensional lattice and x be an arbitrary point of $E^{n}$. If *D* is the Dirichlet–Voronoi cell of Λ, find a lattice point v satisfying x∈D+v.

We end this section with 2 well-known problems about the Dirichlet–Voronoi cells of lattices.

**Problem 4.6.** When n≥6, classify all Dirichlet–Voronoi cells of the *n*-dimensional lattices, i.e., determine their geometric shapes.

**Voronoi’s conjecture.** Every parallelotope is an affine image of a lattice Dirichlet–Voronoi cell.

When n≤5, both Problem 4.6 and Voronoi’s conjecture have been solved. The Dirichlet–Voronoi cell has been applied to lattice-based cryptography by Micciancio and others since 2010.

## Positive Definite Quadratic Forms

Let Λ be a lattice with a basis $\left\{a_{1},\;a_{2},\ldots ,\;a_{n}\right\}$, where $a_{i}=$$\left(a_{i1},\;a_{i2},\ldots ,\;a_{\text{in}}\right)$, and let *A* denote the n×n matrix with entries $a_{\mathrm{ij}}$. Then, the lattice can be expressed as

$$\Lambda =\left\{zA:\;z\in {\mathbb{Z}}^{n}\right\}$$ (51)

and the norms of the lattice vectors can be expressed as a positive definite quadratic form

$$Q\left(z\right)=\langle zA,\;zA\rangle =zAA^{\prime }z^{\prime },$$ (52)

where $A^{\prime }$ and $z^{\prime }$ indicate the transposes of *A* and z, respectively. Assume that

$$Q\left(x\right)=\sum_{1\leq i,j\leq n}c_{\mathrm{ij}}x_{i}x_{j}=xCx^{\prime }$$ (53)

is a positive definite quadratic form of *n* variables, where $c_{\mathrm{ij}}=c_{\mathrm{ji}}$ and *C* is the symmetric matrix with entries $c_{\mathrm{ij}}$. It is known that there is an n×n matrix *A* satisfying $C=AA^{\prime }$. Then, the quadratic form also produces a lattice

$$\Lambda =\left\{zA:\;z\in {\mathbb{Z}}^{n}\right\}.$$ (54)

Therefore, there is a nice correspondence between lattices and positive definite quadratic forms. Then, the SVP is equivalent to the following problem.

**SVP in quadratic form.** Find a nonzero vector $z\in {\mathbb{Z}}^{n}$ that minimizes the positive definite quadratic form $Q\left(z\right)$*.*

Let $\mathrm{dis}\left(Q\right)$ be the discriminant of the quadratic form $Q\left(x\right)$ and let $Q_{n}$ denote the family of all positive definite quadratic forms in *n* variables. Then, we define

$$m\left(Q\right)=\underset{z\in {\mathbb{Z}}^{n}\backslash \left\{o\right\}}{\text{min}}Q\left(z\right)$$ (55)

and

$$\gamma_{n}=\underset{Q\in Q_{n}}{\text{sup}}\frac{m\left(Q\right)}{\sqrt[{n}]{\mathrm{dis}\left(Q\right)}}.$$ (56)

Usually, $\gamma_{n}$ is called Hermite’s constant. These constants are closely related to the densities $\delta^{*}\left(B^{n}\right)$ of the densest lattice ball packings. Since $\ell \left(\Lambda \right)=\sqrt{m\left(Q\right)}$ and $\mathrm{dis}\left(Q\right)=\det{\left(\Lambda \right)}^{2}$, one can easily deduce that

$$\delta^{*}\left(B^{n}\right)=\frac{\omega_{n}\gamma_{n}^{n/2}}{2^{n}},$$ (57)

where $\omega_{n}$ is the volume of the *n*-dimensional unit ball $B^{n}$. In fact, all the known exact results about $\delta^{*}\left(B^{n}\right)$ $\left(\text{except}\;\delta^{*}\left(B^{24}\right)\;\right)$ were derived from the known results about $\gamma_{n}$ (see Table 4).

**Table 4.** Known results about Hermite’s constants

*n*	$\gamma_{n}$	Author Date	*n*	$\gamma_{n}$	Author Date
2	$2/\sqrt{3}$	Lagrange 1773	6	$\sqrt[{6}]{\frac{64}{3}}$	Blichfeldt 1925
3	$\sqrt[{3}]{2}$	Gauss 1831	7	$\sqrt[{7}]{64}$	Blichfeldt 1926
4	$\sqrt{2}$	Zolotarev, Korkin 1872	8	2	Blichfeldt 1934
5	$\sqrt[{5}]{8}$	Zolotarev, Korkin 1877	24	4	Cohn, Kumar 2009

In lattice-based cryptography, approximating SVP is practically important. In fact, both SIS and LWE can be reduced to this type of problems. Let γ be a suitable positive number or a suitable function of *n*. Then, the SVP_γ_ asking, for any given *n*-dimensional lattice Λ, to find a nonzero lattice point v∈Λ satisfying

$$||\;v\;||\leq \gamma \ell \left(\Lambda \right).$$ (58)

In terms of positive definite quadratic forms, the SVP${}_{\gamma }$ can be reformulated as:

**SVPγ in quadratic form.** For a given positive definite quadratic form $Q\left(z\right)$ and a suitable approximation parameter γ, find a nonzero integral solution z to

$$Q\left(z\right)\leq \gamma^{2}m\left(Q\right).$$ (59)

In 1953, R. A. Rankin [51] introduced a generalization of Hermite’s constant. Let *r* be an integer, 1≤r≤n−1, and let $m_{r}\left(Q\right)$ denote the lower bound of any principal minor of order *r* of any form equivalent to $Q\left(x\right)$. He defined

$$\gamma_{n,r}=\underset{Q\in Q_{n}}{\text{sup}}\frac{m_{r}\left(Q\right)}{\mathrm{dis}{\left(Q\right)}^{r/n}}.$$ (60)

Twenty years ago, Rankin’s constant led P. Nguyen and others to introduce the DSP, a generalization of the SVP. This new problem has been studied by Micciancio, Nguyen, and others. It has important applications to blockwise lattice reduction generalizing LLL and Schnorr’s algorithm.

Assume that $\Lambda =\left\{zA:\;z\in {\mathbb{Z}}^{n}\right\}$ is an *n*-dimensional lattice in $E^{n}$, where *A* is a nonsingular n×n matrix. For any point $p=yA\in E^{n}$ and v=zA∈Λ, we have

$$\begin{array}{c} ∥\;p\;-v\;∥=∥\;\left(y-z\right)A\;∥=\sqrt{Q\left(y-z\right)}. \end{array}$$ (61)

Therefore, the CVP is equivalent to the following problem.

**CVP in quadratic form.** Given a positive definite quadratic form $Q\left(x\right)$ and a vector y, find an integer vector $z\in {\mathbb{Z}}^{n}$ that minimizes $Q\left(y-z\right)$.

Let *C* denote the unit cube $\left\{\left(x_{1},\;x_{2},\ldots ,\;x_{n}\right):\;0\leq x_{i}<1\right\}$, let Λ be the lattice corresponding to $Q\left(x\right)$, and define

$$\rho \left(Q\right)=\sqrt{\underset{y\in C}{\text{max}}\;\underset{z\in {\mathbb{Z}}^{n}}{\text{min}}\;Q\left(y-z\right)}.$$ (62)

It can be verified that $\rho \left(Q\right)$ is the smallest number ρ such that $\rho B^{n}+\Lambda$ is a ball covering of $E^{n}$. Consequently, we get

$$\theta^{*}\left(B^{n}\right)=\underset{Q\in Q_{n}}{\text{min}}\frac{\omega_{n}\rho {\left(Q\right)}^{n}}{\sqrt{\mathrm{dis}\left(Q\right)}}.$$ (63)

In fact, most of the known exact results about $\delta^{*}\left(B^{n}\right)$ and $\theta^{*}\left(B^{n}\right)$ were achieved by studying quadratic forms.

Besides the fact that both SVP and CVP can be reformulated in terms of quadratic forms, in recent years Nguyen, L. Ducas, and others have applied quadratic forms directly to lattice-based cryptography.

### Reduction theory of quadratic forms (lattices)

If $\left\{a_{1},\;a_{2},\ldots ,\;a_{n}\right\}$ is an orthogonal basis of Λ, then the corresponding quadratic form $Q\left(x\right)=xCx^{\prime }$ is standard. In this case, both SVP and CVP can be solved easily, since the shortest basis vector is the shortest nonzero lattice vector of Λ. If $w=w_{1}a_{1}+w_{2}a_{2}+\cdots +w_{n}a_{n}\in E^{n}$, taking

$$v=\left\lfloor \left.w_{1}\right\rceil a_{1}+\right.\left\lfloor \left.w_{2}\right\rceil a_{2}+\right.\cdots +\left\lfloor \left.w_{n}\right\rceil a_{n},\right.$$ (64)

where $\left\lfloor \left.x\right\rceil \right.$ denotes the closest integer to *x*, one can show that v∈Λ is a closest lattice vector of w.

It is well known that most lattices have no orthogonal bases. Nevertheless, every lattice has some relatively good bases. Correspondingly, every positive definite quadratic form has a comparatively good equivalent form. This is the philosophy of reduction theory. In history, reduction theory was first developed for quadratic forms rather than for lattices.

Let *U* be a unimodular matrix and write

$$\tilde{Q}\left(x\right)=x\mathrm{UC}U^{\prime }x^{\prime }.$$ (65)

We say $\tilde{Q}\left(x\right)$ is equivalent to $Q\left(x\right)$. Since the map z→zU is an automophism in ${\mathbb{Z}}^{n}$, one has

$$m\left(\tilde{Q}\right)=m\left(Q\right)$$ (66)

and

$$\mathrm{dis}\left(\tilde{Q}\right)=\text{det}\left(\mathrm{UC}U^{\prime }\right)=\mathrm{dis}\left(Q\right).$$ (67)

In 1773, Lagrange proved that every positive definite binary quadratic form $Q\left(x\right)=xCx^{\prime }$ is equivalent to one satisfying

$$\left\{\begin{array}{cc} c_{11}\leq c_{22}, & \;\\ 0\leq 2c_{12}\leq c_{11}, & \; \end{array}\right.$$ (68)

which marked the birth of the reduction theory. In other words, every 2-dimensional lattice has a basis $\left\{a_{1},\;a_{2}\right\}$ such that the angle between $a_{1}$ and $a_{2}$ is at least π/3 and at most π/2. Then, one can deduce that $\gamma_{2}=2/\sqrt{3}$ and $\delta^{*}\left(B^{2}\right)=\pi /\sqrt{12}.$

Reduction theory has been further developed by Seeber, Gauss, Hermite, Korkin, Zolotarev, Minkowski, Voronoi, and many modern authors (see [14,36]). We introduce 3 reductions as examples.

### Korkin–Zolotarev reduction

In 1873, Korkin and Zolotarev proposed the following reduction: A positive definite quadratic form $Q\left(x\right)$ is said to be K–Z reduced if

$$Q\left(x\right)=\sum_{i=1}^{n}c_{i}{\left(x_{i}+\sum_{j=i+1}^{n}t_{\mathrm{ij}}x_{j}\right)}^{2},$$ (69)

where $|\;t_{\mathrm{ij}}\;|\leq 1/2$ and

$$\begin{array}{c} c_{i}=\underset{\left(z_{i},z_{i+1},\ldots ,z_{n}\right)\neq o}{\text{min}}\left\{\sum_{j=i}^{n}c_{j}{\left(z_{j}+\sum_{k=j+1}^{n}t_{\mathrm{jk}}z_{k}\right)}^{2}\right\}. \end{array}$$ (70)

Clearly, the first basis vector in the corresponding lattice of a K–Z reduced form is the shortest nonzero lattice vector. Then, they proved the following theorem.

**Theorem 5.1.** Every positive definite quadratic form is equivalent to a K–Z reduced one.

Korkin and Zolotarev were not able to explore further in this direction since Zolotarev died in 1878 at the age of 31. However, in 1934, Blichfeldt succeeded in determining the values of $\gamma_{6}$, $\gamma_{7}$, and $\gamma_{8}$ by Korkin and Zolotarev’s reduction theory (see [14]). In particular, in 1987, Schnorr [39] developed a generalization of the LLL algorithm based on this reduction, known as block Korkin–Zolotarev (BKZ) algorithm, to approximate the SVP.

### Minkowski reduction

As a generalization of Lagrange’s pioneering work, in 1905, Minkowski discovered the following reduction: As usual, we denote the greatest common divisor of *k* integers $z_{1},z_{2},\ldots ,z_{k}$ by $\gcd\left(z_{1},\;z_{2},\ldots ,\;z_{k}\right)$. A positive definite quadratic form $Q\left(x\right)=xCx^{\prime }$ is said to be Minkowski reduced, if

$$c_{1j}\geq 0,\;j=2,\;3,\ldots ,\;n,$$ (71)

and

$$Q\left(z\right)\geq c_{\mathrm{ii}},\;i=1,2,\ldots ,n,$$ (72)

for all integer vectors $z=\left(z_{1},\;z_{2},\ldots ,\;z_{n}\right)$ such that

$$\text{gcd}\left(z_{i},\;z_{i+1},\ldots ,\;z_{n}\right)=1.$$ (73)

It is easy to see that the first basis vector in the corresponding lattice of a Minkowski reduced form is the shortest nonzero lattice vector. Then, he proved the following theorem.

**Theorem 5.2.** Every positive definite quadratic form is equivalent to a Minkowski reduced one.

Minkowski reduction has been studied by many authors, including B. L. van der Waerden, K. Mahler, and E. S. Barnes, in particular with respect to the orthogonality defect of a lattice, which is also useful in lattice-based cryptography.

### Lenstra–Lenstra–Lovász reduction

Assume that $\left\{a_{1},\;a_{2},\ldots ,\;a_{n}\right\}$ is a basis of an *n*-dimensional lattice Λ. We define the associated Gram–Schmidt orthogonal basis as

$$\begin{array}{c} a_{i}^{*}=a_{i}-\sum_{j<i}\mu_{\mathrm{ij}}a_{j}^{*},\;\text{where}\;\mu_{\mathrm{ij}}=\frac{\langle a_{i},\;a_{j}^{*}\rangle }{\langle a_{j}^{*},\;a_{j}^{*}\rangle }. \end{array}$$ (74)

In 1982, Lenstra, Lenstra Jr., and Lovász [37] introduced the LLL reduction: A basis $\left\{a_{1},\;a_{2},\ldots ,\;a_{n}\right\}$ of an *n*-dimensional lattice Λ is called to be LLL reduced if

$$\begin{array}{c} \left|\mu_{ij}\right|=\frac{\left|\langle a_{i},\;a_{j}^{*}\rangle \right|}{\langle a_{j}^{*},\;a_{j}^{*}\rangle }\leq \frac{1}{2}\;\text{for}\;\mathrm{all}\;1\leq \;j<i\leq \;n\; \end{array}$$ (75)

and

$$\begin{array}{c} ∥\;a_{i}^{*}\;{∥}^{2}\geq \sigma ∥\;a_{i-1}^{*}\;{∥}^{2}\;\text{for}\;\mathrm{all}\;i\;=2,3,\ldots ,n, \end{array}$$ (76)

where

$$\sigma =\frac{1}{4}+{\left(\frac{3}{4}\right)}^{n/\left(n-1\right)}.$$ (77)

This time, there is no guarantee that $a_{1}$ is the shortest nonzero lattice vector of Λ. However, it is an approximating shortest nonzero lattice vector. By inventing an algorithm that always can terminate at an LLL reduced basis in polynomial time, they proved Theorem 3.8.

Reduction theory has played a key role in the security analysis of lattice-based cryptography for the classical computer. Naturally, it will be the key tool for the security analysis of lattice-based cryptography for the quantum computer.

## Summary and Outlook

Quantum computing is widely believed to be a revolutionary new technology. In fact, it is a double-edged sword. If efficient quantum computers can be manufactured in the near future, many of the current cryptosystems will be in danger and post-quantum cryptography will be crucial to the security of our communications. It is possible that better cryptosystems can be invented to deal with quantum computing attacks in the future. Nevertheless, up to now, lattice-based cryptosystems are the best candidates to defend the communication security in the forthcoming quantum computer era.

**Fig. 4.**
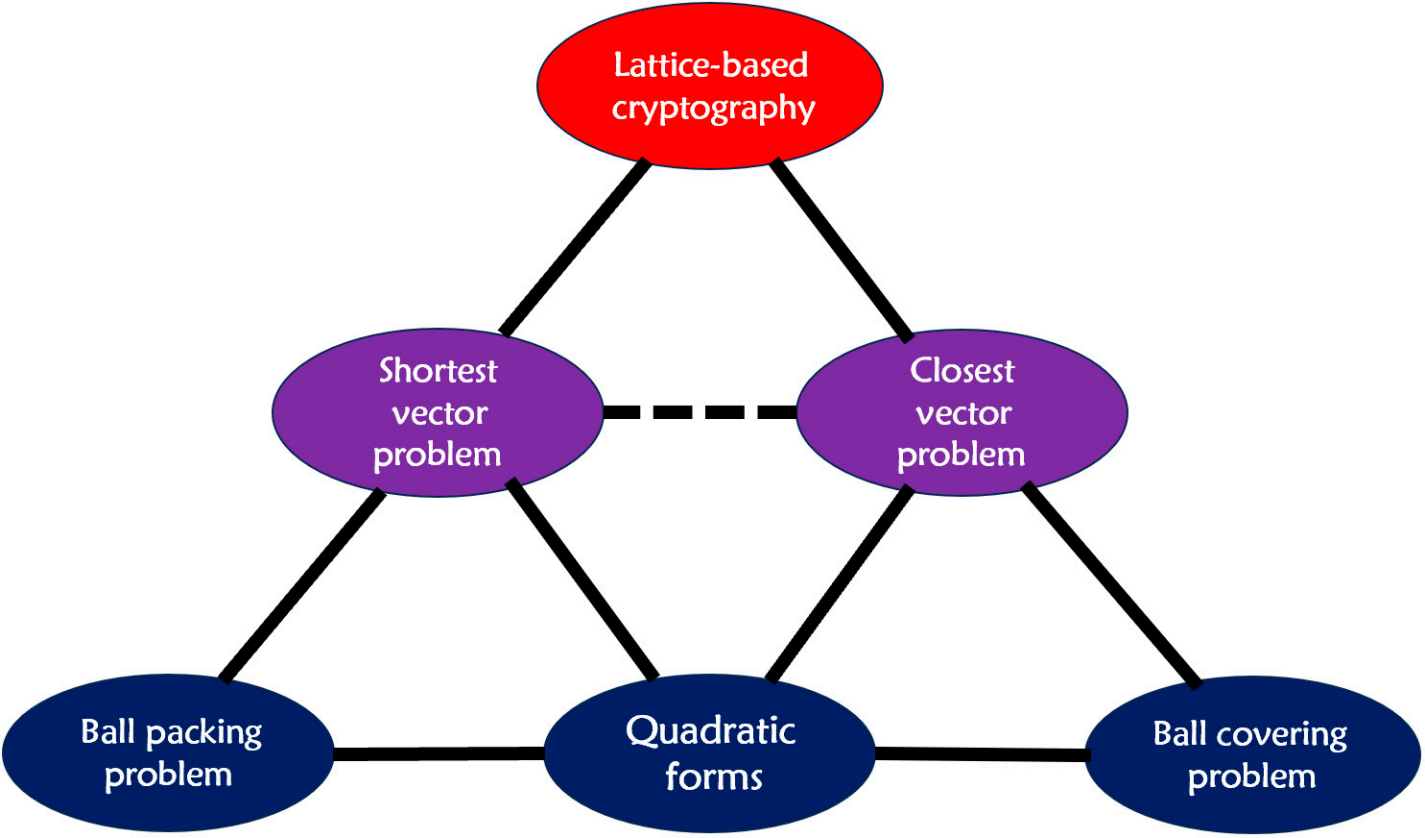
Lattice-based cryptography is deeply rooted in mathematics.

The security of the lattice-based cryptosystems relies on the computational complexity of some fundamental lattice problems such as the SVP, the CVP, and their generalizations, which are deeply rooted in the work of Gauss, Hermite, Korkin, Zolotarev, Minkowski, Siegel, van der Wearden, and many contemporary mathematicians, as shown by Fig. 4. This makes post-quantum cryptography one of the few distinguished examples of crucial modern technology growing up from pure mathematics.

If a new technology can create not only revolutionary progresses but also disastrous harms, preventing the disasters should be much more important and urgent than gaining the benefits. Therefore, post-quantum cryptography provides unprecedented opportunities for mathematicians to make contributions in modern technology (see [52]).

If we compare lattice-based cryptography as a fruit tree, the mathematics discussed in this article should be regarded as its roots. No matter how the post-quantum cryptography will develop in the future, mathematics is inevitable since it needs complicated models just like lattices. Of course, only mathematics is not enough. Successful post-quantum cryptography must be a joint work of mathematicians, cryptographers, and quantum computing scientists.
